# Risk Factors Associated with Falls and Fractures Following Prescription of Opioids Among Privately Insured Patients with Osteoarthritis

**DOI:** 10.36469/001c.32584

**Published:** 2022-08-19

**Authors:** Stuart Silverman, Patricia Schepman, J. Bradford Rice, Craig G. Beck, William Pajerowski, Alan G. White, Sheena Thakkar, Rebecca L. Robinson, Birol Emir

**Affiliations:** 1 Cedars-Sinai Medical Center, Los Angeles, California; David Geffen School of Medicine, University of California Los Angeles; 2 Pfizer Inc., New York; 3 Analysis Group Inc., Boston, Massachusetts; 4 Pfizer Inc., New York, NY; 5 Analysis Group Inc., Boston, MA

**Keywords:** knee osteoarthritis, hip osteoarthritis, analgesic, opioid, tramadol, accidental falls, bone

## Abstract

**Background:** While prior research has shown that patients with osteoarthritis (OA) who are prescribed opioids have higher rates of falls and fractures following drug initiation, there is a limited body of work establishing a comprehensive model of factors that influence the risk of falls or fractures among these patients. **Objective:** Opioids are associated with negative clinical outcomes, including increased risk of falls and fractures. This study assessed the frequency, treatment characteristics, and risk factors associated with falls or fractures among patients with OA taking opioids. **Methods:** Optum Healthcare Solutions, Inc data (January 2012–March 2017) were used to identify patients over 18 with at least 2 diagnoses of hip and/or knee OA, and at least 90 days’ supply of opioids. Patients with cancer were excluded. Falls or fractures outcomes were assessed in the 36-month follow-up period after the date of the first opioid prescription after first OA diagnosis. Demographic, treatment, and clinical characteristics associated with falls or fractures were assessed using logistic regression. **Results:** Of 16 663 patients meeting inclusion criteria, 3886 (23%) had at least 1 fall or fracture during follow-up. Of these 3886 patients, 1349 (35%) had at least 1 fall with an average of 3 fall claims, and 3299 (85%) patients had at least 1 fracture with an average of 8 claims during follow-up. Spine (15.8%) and hip (12.5%) fractures were most common. Median time to fall or fracture was 18.6 and 13.9 months, respectively. Significant (*P*<.05) risk factors associated with at least 1 fall or fracture during the follow-up period included alcohol use (odds ratio [OR], 3.41), history of falling (OR, 2.19), non-tramadol opioid use (OR, 1.31), age (OR, 1.03), benzodiazepine use (OR, 1.21), and at least 1 osteoporosis diagnosis (OR, 2.06). **Discussion:** This study is among only a few that clearly identifies the substantial impact and frequency of falls and fractures associated with prescribing non-tramadol opioids to patients with OA. Findings suggest that fall or fracture risks need to be considered when managing OA pain with opioids. **Conclusion:** Falls and fractures impose a major clinical burden on patients prescribed opioids for OA-related pain management. Falls or fracture risks should be an important consideration in the ongoing treatment of patients with OA.

## INTRODUCTION

Arthritis is the leading cause of disability among adults in the United States; osteoarthritis (OA) is the most common form of arthritis, affecting approximately 32.5 million people worldwide.^1^ OA can cause pain, swelling, and stiffness in patients.^2^ As, unlike other forms of arthritis, there is no approved disease-modifying treatment that slows the progression of the disease, much of the clinical discussion surrounding OA is focused on managing pain and other related symptoms. Pharmacological treatments recommended by the American College of Rheumatology to manage symptoms of OA include topical and oral nonsteroidal anti-inflammatory drugs (NSAIDs), intra-articular glucocorticoid injections, intra-articular hyaluronic acid injections, duloxetine, acetaminophen, tramadol, and non-tramadol opioids.^3^

Although commonly prescribed in management of symptoms associated with OA, opioids are not recommended or only conditionally recommended in certain situations, such as when other treatments have failed.^4^ However, prior research has shown that their usage among patients with OA is more pervasive than the clinical recommendations would suggest.^5,6^ The conditional nature of the recommendation for opioid use by OA patients is based on a number of factors, including the side effects associated with OA use. While risks of addiction, abuse, and misuse are often a main focus of prescribers and patients, other common side effects can include drowsiness and dizziness. These side effects can lead to more serious adverse events, such as falls and subsequent fractures.^7,8^ Although other side effects of opioid prescribing use are well documented, including in patient populations with OA, falls and fractures are equally important clinical outcomes which remain less understood.^9^

Prior research has shown that a substantial proportion of older adults who are prescribed opioids experience falls and fractures following drug initiation.^10–12^ Importantly, falls and fractures represent serious clinical events which impact patients’ quality of life and may even potentially increase mortality risk.^13^ In addition, among patients with OA undergoing total hip replacement, a history of falls prior to surgery has been shown to be associated with worse surgical outcomes, including increased rates of surgical revisions, 30-day and 90-day hospitalizations, postsurgical complications, and higher total healthcare costs (unpublished data).^14^ However, less is known about the effect of opioid use on types and frequencies of falls and fractures among the broader population of patients with OA of the hip and/or knee. Also, regarding the risk factors for falls and/or fractures and in the context of clinical comorbidities, there is limited published research that seeks to establish a comprehensive model of factors that influence fall and fracture risk among elderly patients.^15,16^ Given the gaps in evidence around these common adverse events associated with opioid use, this study sought to assess the frequency, time to event, and risk factors associated with a fall or fracture in a commercially insured US population of patients with OA taking opioids (ie, tramadol or non-tramadol opioids).

This study had 3 main objectives. First, analyses sought to assess and quantify the frequency and other characteristics associated with a fall or fracture among patients with OA of the hip and/or knee using real-world claims data. Next, time to fall or fracture following the prescribing of opioids was assessed using Kaplan-Meier analyses. In addition, this study also sought to develop a comprehensive model of risk factors associated with falls and fractures among patients with OA who were prescribed opioids, including prescribing of non-tramadol opioids, patient comorbidities, and other characteristics.

## METHODS

### Data Source

This study used deidentified administrative claims data from OptumHealth Care Solutions, Inc (Optum), a database containing healthcare utilization records for approximately 19 million privately insured lives (including employees, spouses, dependents, and retirees) from over 84 large, self-insured, US-based companies. The database contains information regarding patient age, gender, enrollment history, medical diagnoses, procedures performed, dates and place of services, prescription drug use, and payment amounts.

### Sample Selection

Patients with at least 2 diagnoses of OA of the hip and/or knee (according to the *International Classification of Diseases, Ninth and Tenth Revision, Clinical Modification* [ICD-9-CM, ICD-10-CM]) and who received at least 1 prescription for an opioid (according to generic product identifier [GPI] codes) during the study period were identified. The list of ICD-9-CM, ICD-10-CM, and GPI codes used are available upon request. The index date was defined as the date of the earliest opioid prescription occurring after each patient’s first OA diagnosis. Patients were required to be at least 18 years old on the index date and be continuously enrolled during the 6 months before (baseline period) and 36 months after (follow-up period) the index date to ensure that all relevant prescription and medical claims were captured. The index date was included in the 36-month follow-up period. Additionally, consistent with prior research on “chronic opioid use” and to help ensure that the prescription opioid(s) were prescribed for OA (as opposed to short-term, acute pain), patients were required to have at least 90 days of cumulative supply of opioids during the follow-up period.^9^ Lastly, patients were divided into 2 mutually exclusive cohorts based on if they had experienced a fall and/or fracture during the follow-up period: a fall/fracture cohort and a no fall/fracture cohort.

### Study Design and Outcomes

This study consisted of a retrospective observational study of privately insured patients with OA in the United States. Characteristics in the baseline period and on index date were described using summary statistics, as were characteristics of falls and fractures in the follow-up period among patients with at least 1 fall or fracture. Next, time to first fall or fracture was assessed using Kaplan-Meier models. Finally, to assess risk of falls and fractures in patients taking opioids, controlling for confounding characteristics, logistic regression was used.

Multiple study outcomes and other variables were considered across analyses. To assess baseline health and sociodemographic characteristics of patients with OA with and without falls/fractures, the following characteristics were summarized at baseline or index date by cohort: age, gender, geographic region, Charlson Comorbidity Index (CCI),^17^ diagnoses of other comorbidities, type of OA diagnoses (eg, hip or knee), and index opioid prescription characteristics. Since physician specialty information is not available from pharmacy data, physician specialty on the most recent medical claim within 2 days of the index date was assessed.

Main study outcomes were defined from primary or secondary diagnoses codes for falls/fractures (ie, ICD-9-CM and ICD-10-CM codes) on the patient’s medical claim. In the 36-month follow-up period, the following characteristics were summarized among patients with at least 1 fall or fracture: rates of falls/fractures and rates of fall/fracture hospitalizations, rates of falls/fractures and hospitalizations by fracture location (ie, hip, spine, and other locations), type (ie, tramadol or non-tramadol opioids; immediate release [IR] or extended release [ER]) and dosage of opioids in the 30 days prior to a fall or fracture, and dosage of sedative-hypnotics in the 30 days prior to a fall or fracture. Fall/fracture hospitalizations were defined based on whether the diagnosis of fall or fracture was identified from an inpatient claim.

Outcomes used in time to event and Kaplan-Meier analysis considered a patient’s first fall/fracture. Time to a patient’s first fall, fracture, and combined fall or fracture, respectively, was assessed using Kaplan-Meier models. In addition to curves showing the percent of patients with an event over time, median time to each event and interquartile range (IQR) for time to each event were shown.

To assess the risk of experiencing a fall or fracture associated with type of opioid drug use and other clinical characteristics, logistic regression models were implemented to estimate odds ratios (ORs) with 95% CIs predicting this risk.

### Statistical Analysis

**Descriptive analysis:** Descriptive statistics (mean, median, SD, percent) were used to summarize and compare baseline characteristics of the 2 cohorts (ie, fall/fracture cohort and no fall/fracture cohort). In addition, descriptive statistics were used to summarize the rates of falls/fractures, falls/fractures leading to hospitalization, and drug use characteristics prior to the first fall or fracture event during the follow-up period, among patients in the fall/fracture cohort.

During the baseline period to compare the two cohorts, statistical significance was assessed using the χ2 test for categorical variables and *t* tests for continuous variables. *P* values at the <.05 level were considered statistically significant. All analyses were conducted using SAS version 9.4 (SAS Institute Inc, Cary, North Carolina).

**Time-to-event analysis:** Kaplan-Meier time-to-event models were used to assess time to first event among patients with a fall or fracture. Due to the sample selection of patients with continuous eligibility and at least 1 fall/fracture, no censoring occurred and all patients in this analysis experienced an event over 36 months.

**Risk factors analysis:** Logistic regression models were used to assess risk factors for patients experiencing 1 or more fall or fracture in the follow-up period. Patients’ relative risk of a fall or fracture was assessed controlling for baseline comorbidities, baseline benzodiazepine use, age, region, OA diagnosis type (ie, hip vs non-hip), type of index opioid (eg, tramadol vs non-tramadol opioids), and index opioid dose (in terms of morphine milligram equivalents [MME]).^18^ Relative risk associated with a particular risk factor was expressed in ORs.

## RESULTS

### Sample Selection

**Figure 1.**
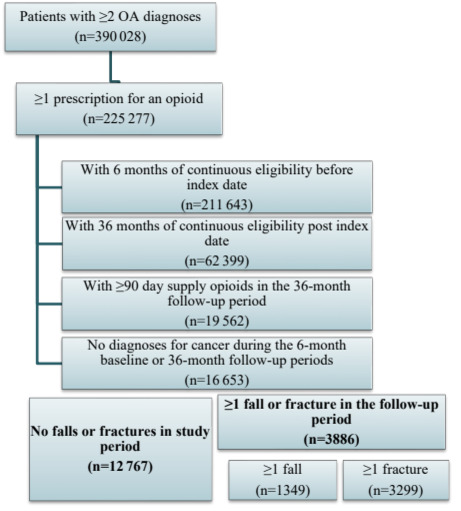
Sample Selection of Cohorts

The inclusion and exclusion criteria and the final sample counts are presented in **Figure 1** by cohort. During the study period, 225383 patients were identified with at least 2 diagnoses of OA of the hip and/or knee who had at least 1 prescription for an opioid. 3886 of 16663 (23%) patients meeting the inclusion/exclusion criteria had at least 1 fall or fracture during the follow-up period (**Figure 1**). Of these patients, 1349 (35%) had at least 1 fall with an average of 1.4 fall claims, and 3299 (85%) patients had at least 1 fracture with an average of 5.3 fracture claims during the follow-up.

### Baseline Characteristics

Patients with a fall or fracture were older on average than patients without a fall or fracture (66.2 vs 61.9 years old, *P*<.01) and also had a higher rate of patients that were female (72.1% vs 61.2%, *P*<.01) (**Table 1**).

**Table 1.** Baseline Characteristics Among OA Patients Prescribed Opioids

**Baseline Characteristics (6 months)**	**Fall/Fracture (n = 3886)**	**No Fall/Fracture (n = 12 767)**	**Relative Difference (%)**	***P* Value**
Age, mean ± SD [median]	66.2± 12.8 [65]	61.9 ± 1.4 [60]	7.0	<.01
≥65, n (%)	1960 (50.4)	4521 (35.4)	42.4	<.01
Gender, male, n (%)	1086 (27.9)	4955 (38.8)	-28.0	<.01
Region, n (%)				
East North Central	1398 (36.0)	4124 (32.3)	11.4	<.01
East South Central	126 (3.2)	465 (3.6)	-11.0	.24
Middle Atlantic	681 (17.5)	2483 (19.4)	-9.9	<.01
Mountain	145 (3.7)	536 (4.2)	-11.1	.20
New England	103 (2.7)	331 (2.6)	2.2	.84
West South Central	251 (6.5)	934 (7.3)	-11.7	.07
Pacific	139 (3.6)	486 (3.8)	-6.0	.51
South Atlantic	338 (8.7)	1379 (10.8)	-19.5	<.01
West North Central	272 (7.0)	864 (6.8)	3.4	.62
Unknown	433 (11.1)	1165 (9.1)	22.1	<.01
Large metropolitan area, n (%)	329 (8.5)	1170 (9.2)	-7.6	.18
Clinical characteristics				
Comorbidities, n (%)				
Acute renal failure	111 (2.9)	145 (1.1)	151.5	<.01
Alcohol dependence	10 (0.3)	19 (0.1)	72.9	.16
Alcohol use	47 (1.2)	53 (0.4)	191.3	<.01
Anxiety	291 (7.5)	630 (4.9)	51.8	<.01
Balance impairment	242 (6.2)	361 (2.8)	120.2	<.01
Chronic kidney disease	210 (5.4)	411 (3.2)	67.9	<.01
Stage 1	6 (0.2)	16 (0.1)	23.2	.66
Stage 2	13 (0.3)	43 (0.3)	-0.7	.98
Stage 3	134 (3.4)	258 (2.0)	70.6	<.01
Stage 4	32 (0.8)	42 (0.3)	150.3	<.01
Stage 5	8 (0.2)	8 (0.1)	228.5	.02
Unspecified	71 (1.8)	124 (1.0)	88.1	<.01
Congestive heart failure	209 (5.4)	307 (2.4)	123.7	<.01
Constipation	162 (4.2)	270 (2.1)	97.1	<.01
Depression	308 (7.9)	666 (5.2)	51.9	<.01
Dialysis	1 (0.0)	3 (0.0)	9.5	1.00
Fatigue	496 (12.8)	1153 (9.0)	41.3	<.01
Glucocorticoid use	13 (0.3)	27 (0.2)	58.2	.17
History of falling	48 (1.2)	35 (0.3)	350.6	<.01
Hypertension	1366 (35.2)	3799 (29.8)	18.1	<.01
Muscle weakness	146 (3.8)	212 (1.7)	126.3	<.01
Myocardial infarction	33 (0.8)	56 (0.4)	93.6	<.01
Nausea	233 (6.0)	432 (3.4)	77.2	<.01
Neuropathy	105 (2.7)	212 (1.7)	62.7	<.01
Osteopenia	278 (7.2)	597 (4.7)	53.0	<.01
Osteoporosis	633 (16.3)	1062 (8.3)	95.8	<.01
Parkinson’s disease	30 (0.8)	33 (0.3)	198.7	<.01
Revascularization	0 (0.0)	15 (0.1)	-100.0	.03
Sleep-related conditions	394 (10.1)	1307 (10.2)	-1.0	.86
Smoking	77 (2.0)	201 (1.6)	25.9	.08
Sedative-hypnotics				
Benzodiazepine	1024 (26.4)	2674 (20.9)	25.8	<.01
Prescription insomnia drug	0 (0.0)	0 (0.0)	-	1.00
Transient ischemic attack	38 (1.0)	83 (0.7)	50.4	.04
Unstable angina	34 (0.9)	81 (0.6)	37.9	.11
Vitamin D deficiency	213 (5.5)	553 (4.3)	26.5	<.01
CCI, mean ± SD [median]	0.6±1.0 [0]	0.0±0.8 [0]	54.5	<.01
OA diagnosis, n (%)				
Hip only	555 (14.3)	1656 (13.0)	10.1	.03
Knee only	1757 (45.2)	6551 (51.3)	-11.9	<.01
Knee and others	645 (16.6)	1734 (13.6)	22.2	<.01
Hip and others	175 (4.5)	458 (3.6)	25.5	<.01
Only hip and knee	116 (3.0)	323 (2.5)	18.0	.12
Hip, knee, and other	55 (1.4)	130 (1.0)	39.0	.04
Index drug characteristics				
Tramadol, n (%)				
IR	865 (22.3)	3345 (26.2)	-15.0	<.01
ER	30 (0.8)	111 (0.9)	-11.2	.56
Non-tramadol opioids, n (%)				
IR	2805 (72.2)	8881 (69.6)	3.8	<.01
ER	421 (10.8)	1,122 (8.8)	23.3	<.01
Index opioid dose (in MME), mean ± SD [median]	60.3±186.9 [30]	51.6±106.5 [30]	16.9	<.01
Physician specialty on index, n (%)				
PCP	793 (20.4)	2586 (20.3)	0.7	.84
Orthopedist	482 (12.4)	1672 (13.1)	-5.3	.26
Rheumatologist	98 (2.5)	320 (2.5)	0.6	.96
Other	1673 (43.1)	4716 (36.9)	16.5	<.01
Missing	1398 (36.0)	5145 (40.3)	-10.7	<.01

Abbreviations: CCI, Charlson Comorbidity Index; ER, extended release; IR, instant release; MME, morphine milligram equivalent; PCP, primary care physician.

Patients with a fall or fracture had higher rates of comorbidities at baseline than patients without a fall or fracture, as measured by CCI (0.6±1.0 vs 0.4±0.8, *P*<.01). In terms of comorbidities related to falls and fractures, patients in the fall/ fracture cohort had higher rates of balance impairment, fatigue, muscle weakness, osteopenia, osteoporosis, and prior falls (all *P*<.01). When considering the location of OA (hip and/or knee with or without OA in other joints), the ordering of prevalence rates per group was consistent across the cohorts, yet the fall or fracture cohort (vs the no fall or fracture cohort) had significantly fewer (*P*<.05) patients with knee only (45.2% vs 51.3%); more patients with knee and other joints (16.6% vs 13.6%), hip only (14.3% vs 13.0%), hip and other joints (4.5% vs 3.6%), and hip, knee, and other joints (1.4% vs 1.0%); and no significant differences in the rate of patients with hip and knee OA without other affected joints (3.0% vs 2.5%).

Patients who experienced a fall or fracture also had different drug utilization characteristics on the index date in comparison with patients without these events. Significantly more patients with a fall or fracture claim received prescriptions for non-tramadol opioids on the index date, both IR (72.2% vs 69.6%, *P*<.01) and ER (10.8% vs 8.8%, *P*<.01), and also had higher index doses in MME (60.3±186.9 vs 51.6±106.5, *P*<.01). Patients with a fall or fracture had similar rates of physician specialty (ie, primary care physician, orthopedist, or rheumatologist) on index date compared with patients without a fall or fracture (**Table 1**).

### Descriptive Characteristics

In the 3-year follow-up period, substantially more patients in the fall/fracture cohort experienced at least 1 fracture (84.9%) than experienced at least 1 recorded fall (34.7%). Among those with at least 1 fall, patients experienced an average of 3.0 (±2.1) falls over the follow-up period, and 27.7% of those falls were associated with a hospitalization. Among the 3299 patients with at least 1 fracture in the follow-up period, the average number of fractures was 8.3 (±11.6), 1052 patients (31.9%) experienced a fracture associated with a hospitalization, and 13.5% of all fractures were associated with a hospitalization (**Table 2**).

**Table 2.** Descriptive Outcomes Among Patients With OA Who Are Prescribed Opioids and With a Fall/Fracture

**Descriptive Outcomes in Follow-up Period (36 months)**	**Fall/Fracture Cohort (n = 3886)**
**Fall/fracture characteristics**	
Falls	
Patients with ≥1 fall, n (%)	1349 (34.7%)
Total number of falls per patient, mean ± SD [median]	3.0 ± 2.1 [2]
Proportion of falls associated with hospitalization, %	27.7%
Fractures	
Patients with ≥1 fracture, n (%)	3299 (84.9%)
Total number of fractures per patient, mean ± SD [median]	8.3 ± 11.6 [4]
Fractures by location	
Hip	
Patients with ≥1 hip fracture, n (%)	485 (12.5%)
Total number of hip fractures per patient, mean ± SD [median]	7.0 ± 7.6 [4]
Spine	
Patients with ≥1 spine fracture, n (%)	613 (15.8%)
Total number of spine fractures per patient, mean ± SD [median]	6.1 ± 7.7 [4]
Non-hip/non-spine	
Patients with ≥1 non-hip/non-spine fracture, n (%)	2820 (72.6%)
Total number of non-hip/non-spine fractures per patient, mean ± SD [median]	7.7 ± 11.1 [4]
Fractures resulting in hospitalization	
All fractures	
Proportion of fractures associated with hospitalization, %	13.5%
Patients with ≥1 fracture associated with hospitalization, n (%)	1052 (27.1%)
Total number of fracture(s) associated with hospitalization per patient, mean ± SD [median]	3.5 ± 4.3 [2]
Hip	
Proportion of hip fractures associated with hospitalization, %	35.7%
Patients with ≥1 hip fracture associated with hospitalization, n (%)	332 (8.5%)
Total number of hip fracture(s) associated with hospitalization per patient, mean ± SD [median]	3.6 ± 3.1 [3]
Spine	
Proportion of spine fractures associated with hospitalization, %	20.4%
Patients with ≥1 spine fracture associated with hospitalization, n (%)	241 (6.2%)
Total number of spine fracture(s) associated with hospitalization per patient, mean ± SD [median]	3.1 ± 2.0 [2]
Non-hip/non-spine	
Proportion of non-hip/non-spine fractures associated with hospitalization, %	12.2%
Patients with ≥1 non-hip/non-spine fracture associated with hospitalization, n (%)	772 (19.9%)
Total number of non-hip/non-spine fracture(s) associated with hospitalization per patient, mean ± SD [median]	3.5 ± 4.4 [3]
**Drug use characteristics**	
Opioid	
Cumulative dose (in MME) in 30 days preceding fall/fracture, mean ± SD [median]	2376.7 ± 12 863.4 [886]
Opioid molecules, ≥1 prescription in 30 days preceding fall/fracture, n (%)	2212 (56.9%)
Tramadol	
IR	488 (12.6%)
ER	24 (0.6%)
Non-tramadol opioids	
IR	1694 (43.6%)
ER	448 (11.5%)
Sedative-hypnotics	
≥1 benzodiazepine prescription in 30 days preceding fall/fracture, n (%)	559 (14.4%)
No. of benzodiazepine prescriptions in 30 days preceding fall/fracture, mean ± SD [median]	1.1 ± 0.4 [1]
≥1 insomnia drug prescription in 30 days preceding fall/fracture, n (%)	0 (0.0%)
No. of insomnia drug prescriptions in 30 days preceding fall/fracture, mean ± SD [median]	—

Abbreviations: ER, extended release; IR, instant release; MME, morphine milligram equivalent.

The most common location of fracture was non-hip/non-spine (72.6%), followed by spine (15.8%), and then hip (12.5%). Patients experienced the greatest number of non-hip/non-spine fractures on average (7.7±11.1), followed by hip (7.0±7.6) and spine (6.1±7.7). Hip fractures tended to be the most severe, as 35.7% of all hip fractures were associated with a hospitalization, followed by spine (20.4%) and non-hip/non-spine (12.2%). However, the largest proportion of patients (19.9%) experienced at least 1 hospitalization associated with a non-hip/non-spine fracture when compared with hip (8.5%) and spine (6.2%) (**Table 2**).

Over half the fall/fracture cohort had at least 1 prescription for an opioid in the 30 days prior to a fall or fracture (56.9%).The most commonly used opioids were IR non-tramadol opioids (43.6%), IR tramadol (12.6%), and ER non-tramadol opioids (11.5%). In the 30 days prior to a fall or fracture, patients had an average cumulative opioid dose of 2376.7 MME (±12 863.4). A small number of patients also had at least 1 prescription for benzodiazepines in the 30-day period prior to fall or fracture (14.4%) (**Table 2**).

### Time to Event

**Figure 2.**
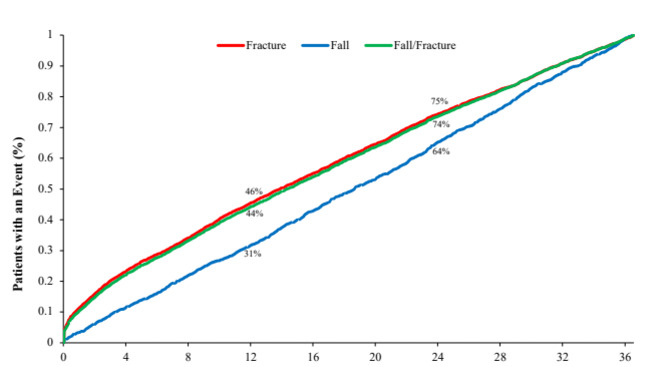
Time from Opioid Initiation to Date of First Fall or Fracture, in Months

Among patients with either a fall or a fracture, the median time to first fall or fracture event was 14.3 months (IQR, 4.9- 24.7). Among patients with a fall, the median time to event was 18.6 months (IQR, 9.2-27.6), and among those with a fracture it was 13.9 months (IQR, 4.6-24.3). At 12 months after the index date, 44% of patients with either a fall or fracture in follow-up had experienced an event, 31% of patients with a fall in follow-up had experienced a fall, and 46% with a fracture had experienced a fracture. At 24 months after the index date, these rates were 74%, 64%, and 75% for the combined fall/fracture outcome, falls alone, and fractures alone, respectively (**Figure 2**).

### Risk Factor Model Results

**Table 3** reports factors identified as associated with a fall or fracture during the 36-month follow-up period. Even controlling for underlying demographics and other baseline characteristics, use of prescriptions of non-tramadol opioids had an estimated OR of 1.31 (*P*<.01), and a dosage increase of 10 MME (ie, patients prescribed higher doses) on index had an estimated OR of 1.01 (*P*<.01).

**Table 3.** Regression Results for Risk Factors Associated With at Least 1 Fall or Fracture During the Follow-up Period

**Risk Factor**	**OR**	**95% CI**	***P* Value**
Age	1.03^a^	(1.023, 1.03)	.00
Gender, male	0.69^a^	(0.638, 0.753)	.00
Region			
East North Central	0.95	(0.776, 1.169)	.64
East South Central	0.97	(0.734, 1.288)	.84
Middle Atlantic	1.01	(0.816, 1.25)	.93
Mountain	0.93	(0.71, 1.223)	.61
New England	1.04	(0.769, 1.402)	.81
West South Central	0.89	(0.697, 1.131)	.33
Pacific (reference group)			
South Atlantic	0.82	(0.648, 1.027)	.08
West North Central	1.12	(0.881, 1.424)	.35
Unknown	1.12	(0.894, 1.405)	.32
Clinical characteristics			
Comorbidities			
Acute renal failure	1.41^a^	(1.063, 1.862)	.02
Alcohol use	3.41^a^	(2.257, 5.153)	.00
Anxiety	1.31^a^	(1.124, 1.539)	.00
Balance impairment	1.40^a^	(1.17, 1.684)	.00
Chronic kidney disease	1.05	(0.859, 1.273)	.66
Congestive heart failure	1.03	(0.821, 1.295)	.79
Constipation	1.31^a^	(1.06, 1.617)	.01
Depression	1.28^a^	(1.096, 1.483)	.00
Fatigue	1.07	(0.947, 1.208)	.28
History of falling	2.19^a^	(1.365, 3.5)	.00
Hypertension	1.05	(0.969, 1.14)	.23
Muscle weakness	1.22	(0.965, 1.544)	.10
Myocardial infarction	1.23	(0.774, 1.941)	.39
Nausea	1.26^a^	(1.056, 1.502)	.01
Neuropathy	1.26	(0.985, 1.619)	.07
Osteopenia^a^	0.69^a^	(0.565, 0.854)	.00
Osteoporosis	2.06^a^	(1.77, 2.396)	.00
Parkinson’s disease	1.90^a^	(1.131, 3.196)	.02
Benzodiazepine use	1.21^a^	(1.109, 1.325)	.00
Transient ischemic attack	0.84	(0.546, 1.28)	.41
Vitamin D deficiency	1.11	(0.935, 1.317)	.23
Charlson Comorbidity Index	1.13^a^	(1.072, 1.188)	.00
OA diagnosis			
Knee (knee only or knee and others) (reference group)			
Hip (only or in combination with other OA)	1.05	(0.961, 1.152)	.27
Index drug characteristics			
Tramadol (reference group)			
Non-tramadol opioids	1.31^a^	(1.199, 1.44)	.00
Index opioid dose (in 10s of MME)	1.01^a^	(1.002, 1.009)	.00

Abbreviations: ER, extended release; IR, instant release; MME, morphine milligram equivalent.

^a^OR is statistically significant at *P*<.05.

Controlling for other baseline characteristics included in the regression, the other clinical characteristics that were significantly (*P*<.05) associated with increased risk of experiencing a fall or fracture were for diagnosis of alcohol use (OR, 3.41), history of falling (OR, 2.19), diagnosis of osteoporosis (OR, 2.06), diagnosis of Parkinson’s disease (OR, 1.90), diagnosis of balance impairment (OR, 1.40), and benzodiazepine use (OR, 1.21). In addition, older age was associated with increased risk (OR, 1.03). Males were at a significantly decreased risk of a fall or fracture (OR, 0.69) compared with females.

## DISCUSSION

The study considered recent, real-world prescribing of opioids to a large sample of commercially insured patients with OA of the hip and/or knee. Opioid use is known to increase the risk of falls and fractures in at least 3 distinct ways, including effects on the central nervous system such as sedation and dizziness, reduced bone density by direct effects on osteoblasts, and hypogonadism induced by chronic opioid use.^19^ Given these effects, the main goal of this study was to assess the frequency, characteristics, and time to event for falls/fractures. In addition, this study also assessed risk factors associated with a fall or fracture in this patient population. At baseline, patients with OA experiencing falls and fractures had significantly higher rates of comorbidities related to falls and fractures, including balance impairment, fatigue, muscle weakness, osteopenia, osteoporosis, and prior falls. Substantial increases were estimated in the frequency of falls/ fractures during the follow-up period after patients were prescribed opioids, highlighting a possible role of drug side effects like drowsiness or dizziness in contributing to adverse events in patients with OA. Among patients with OA meeting the inclusion criteria, approximately 1 in 5 experienced a fall or fracture in the 36-month follow-up period after their first prescription. Study findings also provide additional evidence around the clinical and economic burden of these negative clinical outcomes associated with opioids. For example, 27.7% of all observed falls and 13.5% of all observed fractures were estimated to be associated with an inpatient hospitalization.

Findings from time-to-event analyses highlight the additional need for continuous, long-term observation and clinical management of OA of the hip and/or knee in patients taking opioids by rheumatologists and other clinicians involved in primary care. Among patients experiencing an event, median time to fall and fracture were 18.6, and 13.9 months, respectively. Physicians may increasingly recognize that there is no time point after which patients prescribed opioids for the treatment of OA-related pain are at decreased risk for negative clinical outcomes associated with these medications.^9^

To our knowledge, this study is among only a few that clearly identifies the substantial impact and frequency of falls and fractures associated with prescribing of non-tramadol opioids to patients with OA.^9,20^ We estimate that 57% of patients with OA who were prescribed opioids at index and experiencing a fall or fracture had an opioid prescription in the 30 days prior to the event. The most common type of opioid prescribed during this period were non-tramadol IR opioids, with 44% of fall/fracture patients having at least 1 prescription. These and other findings are supportive of recently updated clinical guidelines from the American College of Rheumatology, which conditionally recommend against prescribing of non-tramadol opioids for patients with hip and knee OA.^21^ Although opioids can help alleviate pain associated with OA, our analysis, based on observed prescription and medical claims, found that patients with OA of the hip and/or knee who were prescribed opioids are at a high risk for falls and fractures over the next 3 years.

Given the substantial clinical burden imposed by a fall or fracture for a patient with OA, risks of falls/fractures should be an important consideration in the ongoing treatment of patients with OA.^22^ Findings from the analysis of risk factors suggest successful clinical management of OA in key patient subgroups, such as patients with comorbid Parkinson’s disease, osteoporosis, or other comorbid conditions, may have benefits in terms of reduced falls and fractures. However, controlling for other characteristics, no significant differences in risk were observed for patients with OA of the hip (alone or in combination with other locations) and OA of the knee (alone or in combination with other, non-hip locations). Drug use characteristics of patients experiencing falls and fractures described in this study (**Table 2**) also highlight that these negative clinical outcomes may be a manifestation of ineffective treatment due to concomitant drug use and switching of therapies.

### Limitations

This analysis, consistent with claims analyses more generally, have certain inherent limitations. Underlying data reflect a privately insured patient population and may not be representative of other payer populations, such as Medicare or Medicaid. Identification of patients is reliant on algorithms and may not be perfectly accurate in selecting patients taking opioids for OA use; despite the 90-day drug use threshold for study inclusion, patients may be taking opioids for other reasons. The analysis cannot ensure that patients are using the treatments for OA itself, although the fact that patients are diagnosed with OA and the use of these treatments after the initial OA diagnosis is suggestive.

Analysis relied on the accuracy of diagnosis codes to identify patients with OA and their comorbidity profiles at baseline. Additionally, diagnosis codes for fall events that resulted in medical interventions, in particular, may lack specificity and may underreport the true prevalence of falls in this population.^23^ Approximately 30% of falls among the elderly are estimated to result in medical intervention.^24^ Any miscoding along these lines will affect the results.

By considering the 3-year period after the patient with OA’s first opioid prescription, results are able to better identify the associated impact of prescription drug treatment independent of any baseline comorbidities. However, certain potential confounding characteristics that may have an effect on risk of experiencing a fall or fracture, such as progression of OA disease severity, were not included, and results from the risk analysis should not be interpreted as causative factors for falls/fractures.

## CONCLUSIONS

This study provides new evidence regarding the frequency, characteristics, and statistically significant risk factors contributing to falls/fractures in patients with OA of the hip and/or knee in the United States. Results provide additional understanding as to the extent of negative clinical outcomes associated with prescription opioid medications, in addition to risks of addiction, abuse, and misuse more commonly considered in clinical assessment. Findings also suggest an ongoing unmet need for non-opioid medications to address OA-related pain in patients without imposing significant risk of falls and fractures. Risks of falls/fractures should be an area of increased focus for physicians in clinical assessment and patient education when managing OA-related pain with opioids.

### Disclosures

S.S. is a paid consultant to Pfizer and Eli Lilly and Company in connection with this study. B.R., A.W., and W.P. are employees of the Analysis Group, who were paid consultants to Pfizer and Eli Lilly and Company for this study and development of this manuscript. R.R. is an employee and stockholder of Eli Lilly and Company. P.S., C.B., B.E., and S.T. are employees of Pfizer with stock and/or stock options.

### Meeting Presentation

This study was presented as a poster at ISPOR and appeared in abstract form in *Value Health.* 2021;24(suppl 1):S138.
